# Clinical application of a rapid microbiological test based on capillary zone electrophoresis to assess local skin infection

**DOI:** 10.1186/1756-0500-4-467

**Published:** 2011-10-30

**Authors:** Jacek Szeliga, Marek Jackowski, Ewa Kłodzińska, Bogusław Buszewski, Wojciech Kupczyk

**Affiliations:** 1 Department of General, Gastroenterological and Oncological Surgery, Collegium Medicum in Bydgoszcz, Nicolaus Copernicus University, ul. Sw. Jozefa 53/59 PL-87-100 Toruń, Poland; 2 Chair of Environmental Chemistry and Bioanalytics, Faculty of Chemistry, Nicolaus Copernicus University, PL-87-100 Toruń, Poland

## Abstract

**Background:**

The basic clinical problem associated with infection treatment is the fact that classic, commonly and routinely used isolation and identification methods are based on long-term processes of a phenotypic analysis of microorganisms. Consequently sometimes, especially in small centres, rapid implementation of antibacterial treatment becomes delayed.

The work presents the initial results of rapid microbiological identification based on an original method of capillary zone electrophoresis (CZE). The study involved the analysis of 78 biological samples from post-operative wounds and trophic ulcers.

**Results:**

The attempt was made to identify individual bacterial species based on characteristic features of electropherograms achieved. Finally, G(+) cocci type bacteria and different G(-) rods were identified with sensitivity of 88.1% and specificity of 100%.

**Conclusions:**

Based on the clinical trials using an electrophoretic technique in the field of microbiological diagnostics of infected exudate from a post-operative wound it can be concluded that it is a rapid and relatively sensitive method for initial identification of infectious pathogens.

## Background

The basic aim of clinical microbiological diagnostics is to determine a microorganism responsible for symptomatic infection as precisely as possible in order to select a method of its targeted elimination. However, the basic clinical problem associated with infection treatment is the fact that classic, commonly and routinely used isolation and identification methods are based on long-term processes of a phenotypic analysis of microorganisms. They require laborious cultures using special media, evaluation of growth and morphology of colonies and single cells, most frequently after a staining procedure. Even a seemingly simple test such as a microscopic analysis can take much time because in some cases extremely long, lasting about 30 minutes microscopic observation of a specially prepared direct specimen under different fields of vision is recommended [1]. However, the most important problems for the classic method are associated with phenotypic variability of bacteria and necessity to detect more and more new strains; consequently, new diagnostic algorithms based on the analysis of new, specific features using biochemical and serological testing (immunofluorescence, immunoenzymatic and agglutination methods) have to be developed. As a result of personal diversity and variability within the same species test results are sometimes not entirely reliable. Finally, these methods are characterised by relatively low sensitivity (the minimum threshold of bacterial cell density is approx. 10^4^/mL) and requirement of a time window (approx. 3 days) to perform them. At times, the identification of a specific strain with phenotypic techniques requires method combinations and even then satisfactory results might not be obtained, as significant variability of some features within a strain itself may be a result of endemic environmental effects. Phenotypic techniques such as phage and bacteriocin typing are a bit more specific since they are used for typing within strains [2,3]. However, there may be cases when within the same species or strain some rearrangements of nucleotide sequences in DNA/RNA genetic structures occur that cannot be detected by conventional testing and that, unfortunately, may condition the formation of strain variants of significantly different clinical importance. The parameter which is the most important from a clinical point of view, namely result reliability and method sensitivity has been rapidly improved as a result of using molecular techniques. Genotypic analysis involving looking for strain-specific sequences of DNA or RNA chains has proven to be independent of a phenotype and its environmental instability. A whole array of molecular methods with different indications in the field of clinical microbiology has been developed, including RFLP (restriction fragment length polymorphism) or different combinations of PCR (*Polymerase Chain Reaction*).

Hopes for the future are associated with the most rapid, dynamic methods allowing to monitor microbiological analysis results in real time. Such methods include RT-PCR (*Real-time PCR*). Nonetheless, as a result of direct comparison between phenotypic and molecular techniques all differences between these two groups might not be noted as the final result of a test may depend on many, sometimes completely independent factors, including e.g. different sample preparation techniques. For example, there are reports of falsely negative results of classic bacterial cultures on saliva samples in microbiological diagnostics of respiratory infections in patients with cystic fibrosis [4]. Specific differences in method sensitivity are also reported with relation to some bacterial species, such as *Pseudomonas aeruginosa*, [5]. There are many phenotypic variants: slow-growing, forming small colonies or auxotrophic that do not grow on classic media, and consequently classic microbiological diagnostics may be extremely ineffective [6,7]. Therefore molecular analysis techniques seem to be a part of optimum microbiological diagnostics as they are rapid, extremely sensitive, precise, repeatable or synchronic - in other words universal, but on the other hand, they have some disadvantages too. First of all, they require specialist laboratories and specialist technicians, who are able to prepare material, analyse it and interpret results. They are expensive. The problem may be partially solved by making these processes automatic; nevertheless, only the largest medical and academic centres can afford such molecular microbiology laboratories.

A simple diagnostic system by Buszewski et al. based on capillary zone electrophoresis for rapid, screening identification of selected aetiological factors for symptomatic infections of post-operative wounds was used in this work. This method has been successfully used many times for the identification of bacteria when microbiologically pure strains were present [8,9]. This type of diagnostics has never been used in direct clinical analysis of non-selected patients with symptoms of surface infection of surgical wound or trophic skin ulceration. All bacteriological tests using capillary zone electrophoresis CZE have been so far carried out under laboratory conditions - on reference strains (from a reference strain catalogue), or on single, pre-processed clinical samples (urine samples or biopsy specimens of tissues) [10]. The only study of bacteriological material collected from an infected wound was carried out for typical infections with Escherichia coli, i.e. targeted application analyses of an instant diagnostic test for the presence of E. coli bacteria in infected wound (to be published soon). This is the first analysis of the method involving an incomparably large study material used in routine microbiological diagnostics of infected wounds and trophic ulcerations under hospital conditions. CZE system was also analysed in terms of its eligibility for direct analysis of bacteriologically heterogeneous material. The basic method characteristics were also evaluated, i.e. its sensitivity and specificity in the conditions of analysis of a non-processed biological sample

## Methods

### Capillary electrophoresis system

A system of measuring equipment combined of the following elements used interchangeably was applied for tests:

• HP^3D^CE system (Agilent Technologies, Waldbronn, Germany) equipped with a diode array detector (DAD) manufactured using diode array technology offering the formation of a constant spectrum of a tested substance in real time. The system was connected to the KAYAK workstation with ChemStation software (Hewlett - Packard) for device control and data acquisition

• Helios-α spectrophotometer (Unicam, Cambridge, UK) for spectrophotometric assays. This is a double-beam UV/VIS radiation spectrometer collecting spectra over the range of 190-1100 nm, with a monochromator Seya Namioka with a matrix diffraction grating (MIG) with 1200 lines per nm, with absorbance repeatability at 1A:>0.005A. It was equipped with a quartz cell, 1 cm long.

• Capillaries were made of fused silica by Composite Metal Services (Worcester, UK)

### Buffer

The TBE buffer made of 4.5 mM Tris/4.5 mM boric acid/0.1 mM EDTA (disodium ethylenediaminetetraacetate) with pH = 8.53, diluted with deionised water at the ratio 8:1 with the addition of 0.2 g PEO/40 mL of the solution (polymer concentration 0.5%) was used in the analysis. This polymer solution was then dispersed in the ultrasound bath for 4 hours at 60°C. The ultimate dilution of TBE to 0.0125% was the last preparation stage for the final electrophoretic solution. This solution was used the most frequently in the analysis.

The following buffers were also used in the initial stage of the experiment:

• TRIS buffer with pH = 8.0 (from 1 mM to 25 mM)

• TRIS buffer with pH = 9.03

• Phosphate buffer with pH = 7.5

### Biological samples

A local Bioethics Committee of Collegium Medicum of Nicolaus Copernicus University in Torun approved the study (No KB677/2009). According to the requirements of IEC, each study participant was provided with a detailed study description and was asked to sign an informed consent form.

Biological samples were prepared individually under aseptic conditions in treatment-dressing rooms at the Department of General, Gastroenterological and Oncological Surgery of Collegium Medicum of Nicolaus Copernicus University in Toruń. 78 subsequent patients diagnosed with post-operative wound infection based on standard clinical symptoms were selected for the study. 78 biological samples in total were collected from patients enrolled into the study. When material was collected aetiological factors of infection were not yet known.

The exudate was collected from an infected wound with a sterile syringe (approx. 0.5 cc) and transferred into a sterile, microbiological transport container. The whole sample was diluted to the volume of 1 cc with sterile water (Aqua pro injectione) - and labelled "sample 1". If the exudate was scarce or too thick the site was washed with 0.5-1.0 cc of sterile water. At the same time a classic wound smear using a microbiological spatula was collected and transferred as control material according to standards valid at the Department to the Department of Microbiology of Voivodship Hospital in Toruń for verification analysis (classic methods of bacteriological diagnostics) - "sample 2".

**Figure 1:**
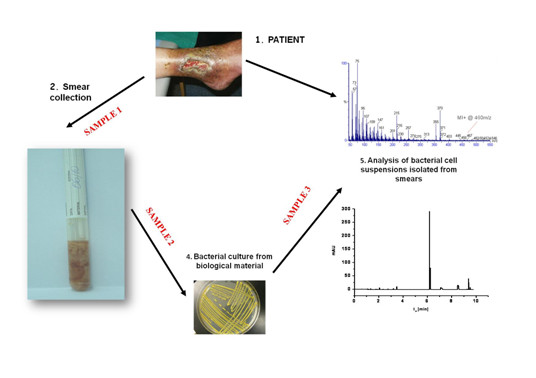
**Algorithm for identification testing**.

The sample 1 was immediately transported to the Chair of Environmental Chemistry and Bioanalytics of UMK in Toruń where it was introduced directly into a CZE-based experimental diagnostic system within 30 minutes. A result in the form of an electropherogram was put together with a phenotypic standard result obtained several days later (sample 2). During the next stage of the experiment a sample was collected under aseptic conditions from the bacteriological medium on the Petri dish where tested bacterial colonies had been cultured and then diluted with water up to 1 cc ("sample 3"). Then this sample was introduced into the CZE system. Consequently, an electropherogram considered to be a standard for microorganisms cultured from the primary biological sample was obtained. The algorithm for a diagnostic strategy is presented on the scheme (Figure 1)

### Preparation methodology for biological samples

A biological sample was injected into the CZE system directly from a transport tube. It was not subject to any preparations beforehand. An important assumption was to minimise time since material collection to analysis.

### Identification of microorganism bands

**Figure 2:**
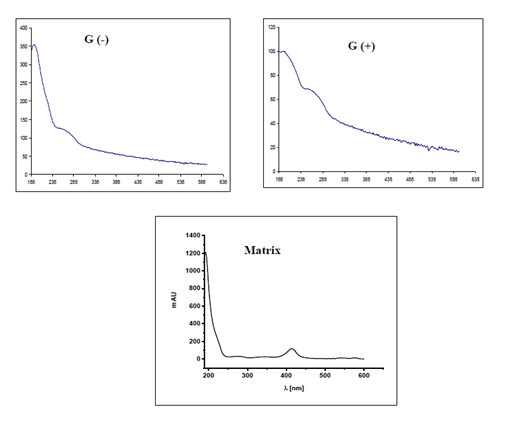
**Characteristic spectra, obtained for different types of bacterial cells and for matrix**.

All signals visible in electropherograms were subject to a qualitative analysis according to the spectral analysis of UV-Vis at λ = 214 nm over the range of 190-600 nm. Characteristic spectra were obtained for different types of bacterial cells and for matrix ingredients present in a sample. Using these spectra, it was possible to identify signals (bands) and to assess tested biological samples qualitatively (Figure 2).

## Results

**Figure 3:**
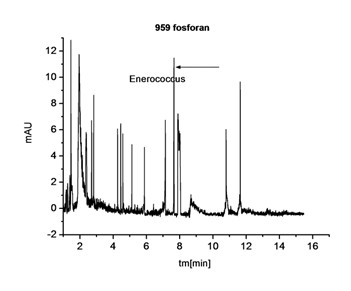
**Electropherogram of a biological smear from a wound in the phosphate buffer without the addition of PEO**.

An electropherogram being an analysis of sample ingredients subject to electrophoresis was a result of each clinical CZE testing. In case of an analysis of a biological sample which was a direct smear from an infected wound it was not possible to exclude the presence of signals coming from bacteria, other organisms (e.g. fungi) and numerous artifacts associated with many organic and cellular ingredients (epithelial and blood cells) present in the exudate. As it turned out when electrophoresis was conducted under inappropriate conditions a completely illegible electropherogram was obtained (Figure 3).

One of the main reasons for this phenomenon was the fact that bacterial cells aggregated in the measuring system and migrated with electroosmotic flow (EOF). Cell aggregations of different sizes were responsible for signals of non-characteristic variable migration in an electric field. As a result of earlier studies by Buszewski et al. and Armstrong using standard bacterial strains the authors knew that one of the elements that could optimise this system was an appropriate buffer, and after the addition of PEO (polyethylene oxide), an antiaggregation agent, the disadvantages above could have been eliminated [10,11]. As a result of using 0.0125% PEO as an additive to the solution it was possible to avoid the phenomenon of mutual aggregation of microorganism cells to the walls of silicon capillaries and to slow down the electroosmotic flow.

**Figure 4:**
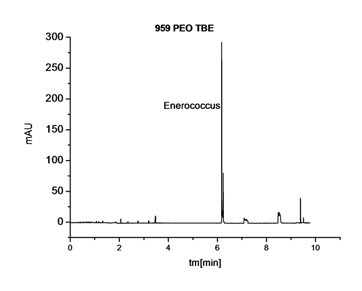
**Electropherogram of the same biological smear from the wound, with the TBE buffer with PEO**.

This buffer ingredient was also effective during an analysis of infected wound exudate. When it was used for the same sample, it was possible to assign signals from bacterial cells unanimously on an electropherogram obtained (Figure 4).

**Figure 5:**
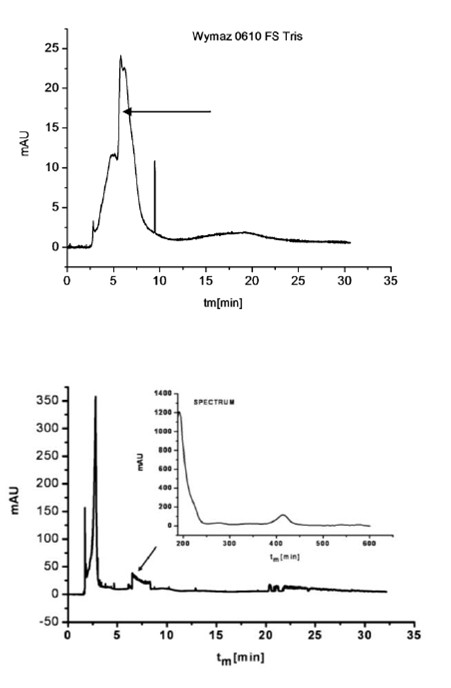
**Background signals in the electropherogram of a biologic sample (matrix effect)**.

As it has already been mentioned, in case of electrophoresis of biological (clinical) material the disadvantageous phenomenon of separation of biological contaminants that are natural ingredients of such exudates (epithelial cells, blood morphotic elements, other organic elements) should be taken into account. However, an electrophoretic analysis of background, namely of a non-infected wound smear (a sample from which no microorganisms were isolated in the verification method) demonstrated that the degree of contamination with biological elements constituting the artifacts mentioned above (so called matrix effect) is low, and it occupies a wide low-intensity band in the electropherogram that migrates with electroosmotic flow (Figure 5). In many cases where bacterial peaks were of a high intensity this phenomenon was simply ignored; however, it depended on the variability of material collected.

When the TBE+PEO buffer with the composition described above was considered as basic (optimum) it was used in all 156 (2*78) tests. Electropherograms of samples 1 and 3 were analysed and verified against the results of a classic analysis of sample 2. Time when a signal appeared, signal intensity and multiplicity were evaluated. Comparable peaks from a biological sample and plate culture (1 and 3) were considered as pathognomic for a specific infection diagnosed with a classic method. For statistical purposes cases of species present as single isolates were rejected (*Stenotrophomonas maltophilia, Klebsiella pneumoniae*) (Table 1).

**Table 1:** Rate of appropriately diagnosed pathogens responsible for post-operative wound infection.

Infection	No	No of CZE diagnoses (%)
*Escherichia coli*	37	32 (86.4)

*Pseudomonas aeruginosa*	4	3 (75.0)

*Enterococcus faecium*	7	

*Streptococcus sp*.	6	22 (100)

*Staphylococcus aureus*	9	

*Klebsiella pneumoniae*	1	0 (0) rejected

*Enterobacter cloacae*	8	6 (75.0)

*Stenotrophomonas maltophilia*	1	1 (100) rejected

Negative	5	5 (100)

TOTAL	76	67 (88,1)

**Figure 6:**
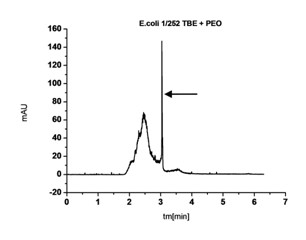
**Electropherogram for *E. coli *bacteria**.

**Figure 7:**
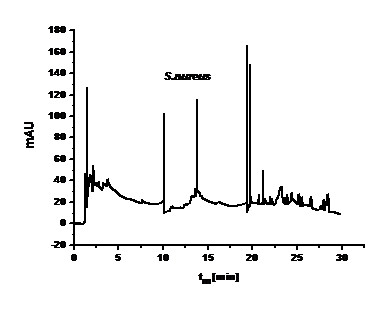
**Electropherogram for staphylococci**.

**Figure 8:**
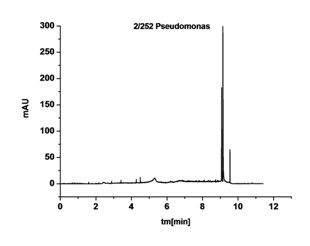
**Electropherogram for *Pseudomonas aeruginosa***.

**Figure 9:**
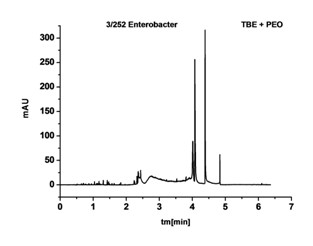
**Electropherogram for *Enterobacter cloacae***.

As a result of this analysis, it was possible to determine the following infections in biological samples (Table 1):

The most characteristic electropherograms, possibly due to a relatively low number of groups, were the ones for the infection caused by:

- *Escherichia coli *that presented signals of the least variability. It was visible in the form of 1 distinctive signal at time t in the compartment from approx. t_m _= 2 to 3 min. (Figure 6)

- multi-peak, irregular signals of a varied intensity, derived from naturally forming cell clusters were typical for G(+) staphylococci (Figure 7)

- *Pseudomonas aeruginosa *(Figure 8) presenting a signal characteristic for all rods, in the form of one, narrow cluster of signals in the time interval of t_m _= 8-10 min.

- *Enterobacter cloacae *presenting, similarly to rods, a cluster of signals in the time interval of t_m _= 4-5 min (Figure 9)

**Figure 10:**
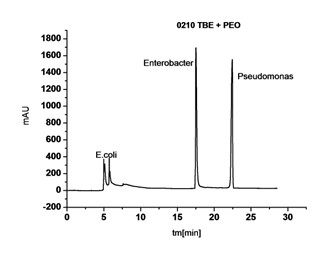
**Electropherogram for the bacterial mixture**. Time intervals when peaks appear are different from the ones for isolates.

Unfortunately, as a result of repeated infection in a biological sample peaks occurred at different time intervals when comparing to signals coming from isolated cultures of individual microorganisms (Figure 10). It was probably related to mutual interactions between different microorganism species what had already been determined by Buszewski et al. in previous studies [10].

An example of such a situation was the analysis of the mixture containing *E. coli*, *Enterobacter cloacae *and *Pseudomonas aeruginosa *- the isolate peaks occurred at different delayed time intervals (Figure 10). However, the order of migration of individual bacterial species remains the same.

**Figure 11:**
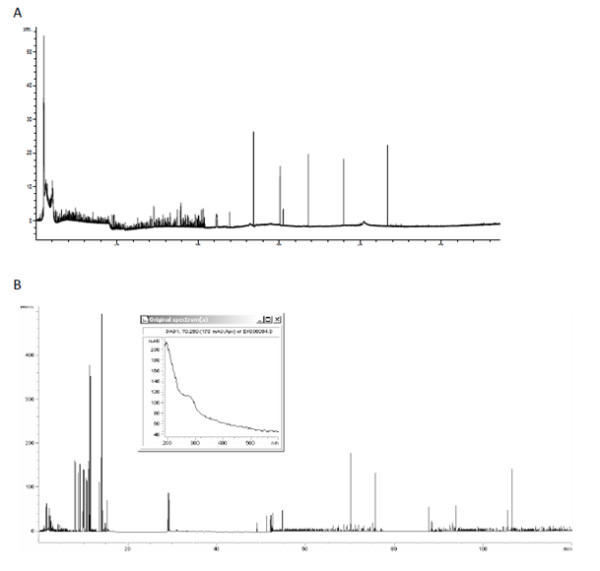
**Electropherogram for *Streptococcus sp*. (A) and *Enterococcus faecium *(B)**.

During further tests on subsequent species it turned out that bacterial species of similar cell morphology present similar signals on electropherograms. Cocci were the most characteristic example for this phenomenon as their electropherogram presented many multiple peaks along the whole axis of time for migration. Bacteria belonging to *Enterococcus*, *Staphylococcus *and *Streptococcus *turned out to be indistinguishable using CZE (Figure 11).

**Figure 12:**
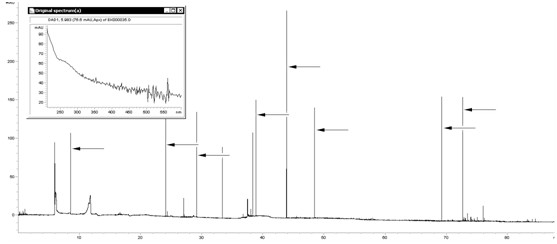
**Electropherogram for a biological sample found sterile in the classic examination**. Many high peaks typical for cocci (arrows) and a wide peak of biological sample background (the matrix effect) are visible.

**Figure 13:**
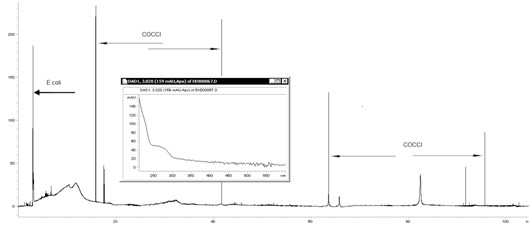
**A sample detection of additional strains in a biological sample in which only *E. coli *was detected using a conventional method**.

An interesting situation was observed during the analysis of a post-operative wound in patient no. 50 who had had laparotomy due to sigmoid perforation. Initially no microorganisms were isolated in the classical method from sample 2 collected from a post-appendectomy wound; however, parallel sample 1 in the CZE analysis revealed a number of peaks that could be typical for bacterial colonies of cocci morphology G(+) (Figure 12). A repeated analysis of serum exudate from this wound demonstrated infection cause by *Staphylococcus epidermidis*. (Figure 12). It may indicate significantly higher sensitivity of the electromigration method comparing to traditional diagnostic techniques. Similarly, in the group of 30 isolated (diagnosed as a result of phenotypic techniques) infections of *E. coli *that were subject to final analysis in 3 cases the CZE spectrum turned out to be ambiguous, because based on its character it was possible to diagnose a complex infection caused by *E. coli *and cluster-forming cocci with a great likelihood. Therefore on the next day classic cultures using material collected from these 3 wounds were repeated. In all 3 cases the repeated results indicated coinfection with *Staphylococcus sp*. (2) and *Enterococcus sp *(1) strains (Figure 13).

Finally, the general sensitivity of the method was determined at the level of 88.1% with specificity of 100%.

## Discussion

Identification of a pathogen causing an infectious disease, local or generalised infection is a basic condition to institute effective treatment. There are cases where traditional culture techniques are fully sufficient to conduct a diagnostic process and there is no need to use more sophisticated methods of microbiological diagnostics. Such situations are present in case of moderately intensified infections of soft tissues, skin, wound suppuration or urinary tract infection. Much worse cases are when infection progresses rapidly and its consequences develop violently and are fatal. An example of situations where infection confirmation has to be done as quickly as possible is intraoperative differentiation of different organ inflammations and mainly septic shocks (sepsis) requiring a distinct, more individualised strategy. In the treatment of such diseases as generalised infection of the body, blood or meningitis institution of an effective antibacterial agent as quickly as possible is the most important parameter. However, it is necessary to confirm the presence of specific pathogenic microorganisms first.

Management standards used so far in such cases include introduction of an antibacterial agent with as broad as possible spectrum of action and a classic anti-shock management strategy combined with waiting for an antibiogram. Unfortunately problems associated with precise identification of an infection focus and delays obtaining a final microbiological diagnosis when body catabolic reactions are very dynamic are often responsible for failure of treatment applied.

The method of rapid microbiological diagnostics based on capillary zone electrophoresis suggested a few years ago by Buszewski et al. hopefully seems to be at least a partial solution to this problem. It indeed does not offer precise identification of every pathogen but as a result of initial identification it is possible to confirm microorganism presence and to limit the spectrum of antibacterial treatment. Based on our material it was possible to determine CZE identification features of such pathogens as: cocci G(+) and different species of rods G(-). Moreover, this method is characterised by ideal specificity (100%).

It can be concluded that this identification technique has several unquestionable advantages over traditional phenotypic typing:

- direct analysis of a biological sample. When the CZE system is used contaminated material may be introduced directly into the system. No sample preparation is necessary. Consequently, a relation between a clinician and a microbiological diagnostician is eliminated and the risk of a so called pre-analytical error is ruled out, and the analysis can be performed in the patient's vicinity. It is more so probable because the equipment is of relatively small dimensions and can be placed in any typical treatment-dressing room.

- short time of analysis. A basic result of a microbiological test using CZE namely electropherogram is achieved as early as after 15-30 minutes. The result can be interpreted in many ways. Even direct visual assessment of peak shapes and time when they appear may provide a relative reliable result as it is in the case of infection by rods G(-) or cocci G(+).

- sensitivity and repeatability. In the group of patients with post-operative wound infection who participated in the study general sensitivity of the method was at the level of 88.1% even when bacteria of different species were mixed. If stable testing parameters are preserved this method is fully repeatable. What is more, there were several cases this method made it possible to detect bacterial colonies in a sample and to deny the results of routine culture (phenotypic) testing and outpace them. At this stage method parameters are limited to selected species and bacterial cell morphology mainly due to density of the population tested. Therefore, further studies have to be conducted.

An important problem that has been observed during studies which slightly decreases a diagnostic value of this method is a phenomenon of interspecies interactions between different microorganisms, the consequence of which is the fact that pathognomic signals in the electropherogram of a bacteriologically complex sample appear at different times than the ones typical for a colony being isolated. So far it is not possible to calculate this phenomenon mathematically; however, based on own observation it can be seen that in almost each case this time was significantly delayed.

Based on a short programme of method clinical application that has been conducted it can be concluded that the diagnostic potential of CZE as a diagnostic method to detect microorganisms has been proven; however, its identification value has to be polished up. The method itself, although combined with other analytical techniques, is a proven test for early rapid qualitative diagnostics of infection. Shin et al. achieved very promising results using PCR combined with CE-SSCP - they isolated 16S rRNA and rDNA from live bacterial cells from their mixture [12]. Unfortunately, this type of assays requires complex and expensive diagnostic equipment.

Our studies were aimed to create a screening algorithm to detect infections rapidly, screeningly, qualitatively and quantitatively based only on the behaviour of bacterial cells during electrophoresis. It was shown to be possible based on initial results of clinical material. It can be proven even by infection caused by Escherichia coli that was detected with the sensitivity of 86.4% when the buffer was selected appropriately. Law et al. used microchip technology and analysis of laser-induced fluorescence to identify the presence of E. coli cells successfully and demonstrated very good parameters of a screening method based on electrophoresis aimed at infections caused by this type of bacteria. Unfortunately each of the samples tested had to be prepared specially for the method to be repetitive [13]. In the method used in our study this stage of analysis was completely omitted and a biological sample was directly introduced into the system. Undoubtedly it makes the whole procedure simpler and no tests conducted by additional specialists are necessary. As it turned out a background signal derived from non-bacterial biological substances was constant, repeatable and in majority of cases insignificant for the analysis.

Relatively high method sensitivity in the determination of simple infections caused by E. coli (86.7%) is an extremely optimistic fact. Homogeneity and repeatability of a signal characteristic for these bacteria was the highest for the universal TBE+PEO buffer. These bacteria were identified earlier based only on a characteristic electrophoretic peak in almost in every sample where the presence of E. coli was later detected. It is possible that such a system will encourage scientists to develop optimum buffers for each bacterial species and to develop a system where biological samples will be processed by a series of parallel capillaries with different buffer solutions. Studies using microchip technology with targeted identification skills towards a specific species or bacterial strain have already been conducted [13].

## Conclusions

Based on the clinical trials using an electrophoretic technique in the field of microbiological diagnostics of infected exudate from a post-operative wound it can be concluded that it is a rapid and relatively sensitive method for initial identification of infectious pathogens. In future, it may become a perfect screening test to diagnose causes of severe generalised infections, consequently, it will be possible to institute first-line antibiotic therapy more precisely (a more narrow spectrum) and in addition, it may be part of infection prophylaxis in the form of industrial contamination analyses of e.g. food products.

## Availability of supporting data

The data sets supporting the results of this article are included within the article.

## List of abbreviations

CZE: capillary zone electrophoresis; IEC: Independent Ethics Committee.

## Competing interests

The authors declare that they have no competing interests.

## Authors' contributions

JS participated in design of the study, took biological samples, performed interpretation of the results, drafted the manuscript, MJ - supervisor of medical part of studies, participated in design of the study, helped to draft the manuscript, WK took biological samples, EK participated in design of the study, carried out biochemical (CZE) studies, performed interpretation of the results, helped to draft the manuscript and BB - supervisor of biochemical studies, participated in design of the study, helped to draft the manuscript

All authors read and approved the final manuscript
